# Stereotypical and Actual Associations of Breast Size with Mating-Relevant Traits

**DOI:** 10.1007/s10508-019-1464-z

**Published:** 2019-09-27

**Authors:** Krzysztof Kościński, Rafał Makarewicz, Zbigniew Bartoszewicz

**Affiliations:** 1 Department of Human Evolutionary Ecology, Faculty of Biology, Adam Mickiewicz University, Umultowska 89, 61-614 Poznan, Poland; 2 Department of Internal Medicine and Endocrinology, Medical University of Warsaw, Warsaw, Poland

**Keywords:** Breast size, Physical attractiveness, Sexual selection, Biological signal, Social perception

## Abstract

Breast size varies substantially among women and influences perception of the woman by other people with regard to her attractiveness and other characteristics that are important in social contexts, including mating. The theory of sexual selection predicts that physical criteria of partner selection should be markers of the candidate’s desirable properties, mainly biological quality. Few studies, however, have examined whether breast size really signals biological quality or its components and whether observers accurately interpret these signals. Our first study encompassed 163 young women and aimed to establish actual correlates of breast size. The aim of the second study was to determine preferences and stereotypes related to breast size: 252–265 women and men evaluated female digital figures varying in, among other characteristics, breast size. Breast size (breast circumference minus chest circumference) was negatively associated with body asymmetry and positively associated with infections of the respiratory system, but did not correlate with infections of the digestive system, openness to casual sex, and testosterone and estradiol level. Women and men perceived breasts in a similar way to each other: the bigger the breasts the higher the reproductive efficiency, lactational efficiency, sexual desire, and promiscuity attributed to the woman. Nevertheless, large breasts were not regarded more attractive than average ones, though small breasts were the least attractive. In addition, big-breasted women were perceived as less faithful and less intelligent than women with average or small breasts. We discuss our results from the perspectives of evolutionary psychology, perceptual biases, and social stereotypes.

**Electronic supplementary material:**

The online version of this article (10.1007/s10508-019-1464-z) contains supplementary material, which is available to authorized users.

## Introduction

Women’s breasts are important in interpersonal relations as they draw attention and arouse sexually (Dixson, Grimshaw, Linklater, & Dixson, 2011a; Schmidt & Sigusch, 1970), even in traditional societies (Eibl-Eibesfeldt, 1989, p. 251; Marlowe, 1998). Breast size impacts on the woman’s physical attractiveness (see below), is heritable (Eriksson et al., 2012; Wade, Zhu, & Martin, 2010), and is highly variable between individuals with the coefficient of variation about 60% (Kayar et al., 2011; Vandeweyer & Hertens, 2002) which is a characteristic for sexually selected traits (Pomiankowski & Møller, 1995). Taken together, these properties suggest that the evolution of breast size has, at least to some extent, been driven by sexual selection.

On grounds of the theory of sexual selection and evolution of biological signals, one can expect that the size of breasts most attractive for men will correspond with the size associated with the highest mate value, or maximum expected reproductive success for a man who mates with the woman (Gangestad & Scheyd, 2005; Sugiyama, 2005). The preference for the breast size signaling the highest mate value would enhance male reproductive success via partner choice. In addition, breast size would evolve toward the value most preferred by men given that this specific breast size makes a woman more attractive to men and competitive in the mating market. However, such evolution may be hampered by other factors, e.g., costs of developing and maintaining body tissues (Gangestad & Scheyd, 2005; Zahavi, 1975), biomechanical effects of breast mass (Kerrigan et al., 2001; Spector & Karp, 2007), and the minimum amount of glandular tissue required to effectively nurse the infant (Linzell, 1972; Neifert, 2001). To assess whether the most attractive size of breasts corresponds with the size associated with the highest mate value, one has to determine which breast size is perceived as the most attractive and how the breast size is related to important components of Darwinian fitness, such as health or lactational capability.

Attractiveness of women with regard to breast size has been extensively investigated. Although the results of many older studies, which applied weakly realistic, schematic stimuli, were divergent (e.g., Furnham, Dias, & McClellan, 1998; Furnham, Swami, & Shah, 2006; Gitter, Lomranz, Saxe, & Bar-Tal, 1983; Singh & Young, 1995; Wiggins, Wiggins, & Conger, 1968), recent research, relying on photographs or images of nearly photographic quality, showed that the most attractive breasts are of average or somewhat above-average size but not very big (Dixson, Grimshaw, Linklater, & Dixson, 2011b; Dixson, Duncan, & Dixson, 2015; Gründl, Eisenmann-Klein, & Prantl, 2009; Swami & Tovée, 2013a, b; Zelazniewicz & Pawlowski, 2011). However, one problem pertaining to all these studies was a lack of stimulus calibration, or more specifically, that particular variants of breast size were prepared without reference to the actual distribution of this trait in the population. Therefore, it is not known which, if any, variant from among those used in the study depicted breasts of average size or how distant from the mean the other variants were (in terms of standard deviations or percentiles). Only one series of studies endeavored to prepare properly average breasts (Dixson et al., 2011a, b, 2015); however, it is not clear whether they succeeded as they relied on British anthropometric standards but assessed preferences in New Zealand, and it is known that breast size depends substantially on genetic ancestry (Chen et al., 2004) and country (“Brit girls’ boobs,” 2007).

Much less is known about whether breast size is a cue to Darwinian fitness or its components. Some studies found that breast size a few days after parturition was positively related to milk production (Hytten, 1954; Lussky, 1951; but see Ramsay, Kent, Hartmann, & Hartmann, 2005), but other researchers regarded these results as an artifact arising from the fact that bigger breasts can store more milk (Daly & Hartmann, 1995). The pace of milk production is regulated by frequency and degree of breast emptying (Daly & Hartmann, 1995) and can increase considerably (with no loss in milk quality) in the case of, for example, breastfeeding of twins (Saint, Maggiore, & Hartmann, 1986) or intensive expression for commercial purposes (Macy, Hunscher, Donelson, & Nims, 1930). In undernourished societies, women produce milk of quantity and quality similar to those observed in rich countries and can successfully breastfeed twins; in addition, one breast can double its output in case of a dysfunction of the other (Prentice et al., 1986). These data suggest that breast size is not a limiting factor of milk production and was not in poorly nourished human ancestors.

As regards associations between breast size and other components of Darwinian fitness, Jasieńska, Ziomkiewicz, Ellison, Lipson, and Thune (2004) reported that women with bigger breasts have higher level of estradiol, which may indicate better reproductive health. However, Garver-Apgar, Eaton, Tybur, and Emery Thompson (2011) and Grillot, Simmons, Lukaszewski, and Roney (2014) did not confirm this relationship, neither did they find an association between breast size and testosterone level—a predictor of poor reproductive health in women (Steinberger, Smith, Tcholakian, & Rodriguez-Rigau, 1979). A low level of body asymmetry is commonly regarded as a cue to high developmental stability, and thereby high genetic or biological quality (Gangestad & Thornhill, 1999; Van Dongen & Gangestad, 2011). Breast size is negatively associated with relative breast asymmetry (Manning, Scutt, Whitehouse, & Leinster, 1997; Møller, Soler, & Thornhill, 1995), but it is not known whether it is related to asymmetry of other body parts. With regard to health, bigger breast size was found to be a risk factor for development of breast cancer (Jansen, Backstein, & Brown, 2014) and type 2 diabetes (Ray, Mohllajee, van Dam, & Michels, 2008). Although previous studies reported infectious health to be correlated with some morphological characteristics, including high facial attractiveness, symmetry, sex-typicality (Gray & Boothroyd, 2012; Shackelford & Larsen, 1997; Thornhill & Gangestad, 2006), and normal body mass (Rantala et al., 2013), its association with breast size has thus far not been examined.

Breast size impacts on male behavior toward the woman: big-breasted women get a hitch-hiking ride more easily, but only when the driver is a man (Guéguen, 2007a), are solicited in night clubs and bars more frequently (Guéguen, 2007b), and big-breasted waitresses obtain higher tips (Lynn, 2009). There is, therefore, a disparity between the preference for average breasts as observed in laboratory studies and the behavioral preference observed in field studies. One possible explanation for this is that big-breasted women are perceived to be more open to casual sex, which encourages men to try initiate a short-term sexual relationship with them. Furnham et al. (1998) found that women with large breasts are perceived as more open to short-term relationships than small-breasted females. However, this study used low-quality line-drawings as stimuli and lacked a version with average breasts. Therefore, it is not known how women with large breasts are perceived compared with those having average breasts.

A related issue is whether breast size is associated with actual openness to casual sex. Smith (1984) proposed that in human ancestors relatively big breasts signaled that the woman is within her lactation period and is therefore temporarily infertile. Since infertile woman cannot be fertilized by a possible lover, the sight of large breasts weakened the vigilance of the woman’s stable partner. In other words, an ancestral man guarded his partner more strongly if she had small breasts and appeared currently fertile than when she had large breasts and appeared to be lactating and currently infertile. Smith hypothesized that ancestral women evolved permanently large breasts in order to effectively cheat on the stable partner. Women differ markedly from each other in breast size (Kayar et al., 2011; Vandeweyer & Hertens, 2002) and openness to casual sex (Penke & Asendorpf, 2008), and each trait depends substantially on genes (Bailey, Kirk, Zhu, Dunne, & Martin, 2000; Eriksson et al., 2012; Wade et al., 2010). If Smith’s hypothesis is correct, it is possible that women’s sexual strategy is related to the breast size because permanently large breasts, which are energetically costly, were profitable only for those ancestral women who were inclined to cheat on their steady partner. One can thus expect that among contemporary women breast size would be positively correlated with openness to casual sex, but this prediction has not been empirically tested.

Previous studies established that women with large breasts are perceived as being older (Dixson et al., 2015; Singh & Young, 1995), sexually more mature, possessing of better reproductive health and nurturing ability (Dixson et al., 2015), but lower in intelligence and morality (Kleinke & Staneski, 1980; Thompson & Tantleff, 1992). However, only the study by Dixson et al. applied stimuli which were of high quality and depicted breasts of small, moderate, and large size. There is, therefore, a requirement for more studies on the perception of mating-relevant traits in relation to the woman’s breast size specifically using stimuli calibrated for distribution of breast size in the population.

To fill some of the above-mentioned knowledge gaps, we conducted two studies. The first was carried out on women and aimed to determine actual correlates of breast size. We checked for linear and nonlinear relationships between breast size and the woman’s body asymmetry, infectious health, estradiol and testosterone level, and openness to casual sex. The second study aimed to establish preferences and stereotypes concerning breast size. Men and women were presented with digital models of women who differed in breast size and asked to assess their physical, sexual, and marital attractiveness, reproductive and lactational capability, sexual desire, faithfulness, intelligence, diligence, and openness to casual sex. Breast size in these figures was manufactured on the basis of anthropometric measurements of real women and a photograph of a topless woman who had average breasts. Many studies showed that men and women are very similar to each other in perception of physical attractiveness, including attractiveness of faces (Henss, 1991), body size (Kościński, 2013), and breasts (Dixson et al., 2015). This similarity may facilitate adaptive modifications of mating behavior and, in humans, own appearance (Brewer, Archer, & Manning, 2007). For this reason, female figures in the second study were assessed by both men and women.

## Study 1

### Method

#### Participants

The study involved 163 young, non-pregnant, non-lactating, Caucasian women (Table 1). These women were students at several schools in Poznań (western Poland), a city being nearly homogenous ethnically. Women were recruited opportunistically and examined at their dwelling place, usually a dormitory.

**Table 1:** Descriptive statistics for women participating in Study 1 and Pearson correlations with breast size

	*n*	*M*	SD	*r*	*p* value
Age (years)	163	22.29	2.59	.07	.398
Height (cm)	163	165.47	5.71	.07	.369
Weight (kg)	163	59.92	9.31	.19	.015
Body mass index	163	21.90	3.39	.16	.045
Breast girth (cm)	163	88.41	7.12	.40	< .001
Chest girth (cm)	163	75.94	6.53	− .02	.841
Breast size (cm)	163	12.47	2.94	–	–
Total Asymmetry	162	0.00	0.74	− .17	.028
Facial Asymmetry	162	− 0.34	0.45	− .11	.165
Hand Asymmetry	162	0.79	0.49	− .14	.068
Respiratory infections	163	0.00	1.00	.21	.008
Count^a^	163	10.61	13.63	.03	.666
Duration (days)^a^	163	6.09	3.36	.19	.016
Antibiotic use	163	0.26	0.29	.25	.002
Digestive infections	163	0.00	1.00	− .02	.774
Count^a^	163	1.18	2.15	− .03	.664
Duration (days)^a^	163	1.62	2.21	.02	.823
Antibiotic use	163	0.12	0.24	− .05	.511
Sociosexual orientation	153	17.51	6.00	− .02	.824
Behavior	156	5.31	2.15	− .06	.436
Attitude	160	6.44	3.13	.00	.982
Desire	157	5.67	2.16	− .03	.700
Saliva estradiol (pg/ml)	74	5.0	2.0	.11	.332
Saliva testosterone (pg/ml)^a^	78	41.2	25.7	.05	.653

^a^Descriptive statistics provided for raw data, correlation with breast size stands for log-transformed data

#### Measures

Assessment of each participant involved the taking of anthropometric measurements, filling out of questionnaires, photography of the face and hands for estimation of body asymmetry, and collection of saliva for hormonal assessment. Participants’ stature was measured with a stadiometer, body mass with scales, and circumference of hips, waist, and the trunk at the level of maximally protruding breasts and just below the breasts, with a measuring tape. We refer to the last two measurements as breast and chest circumference, respectively. Susceptibility to infectious diseases was estimated with a questionnaire that inquired into the number and duration of infectious diseases of the respiratory system (e.g., cold, influenza) and digestive system (e.g., stomach or intestinal flu) in the past 3 years, and the frequency of antibiotic use for both types of infection (Thornhill & Gangestad, 2006).

Openness to casual sex, or sociosexual orientation, was determined with the 9-item Revised Sociosexual Orientation Inventory developed by Penke and Asendorpf (2008, http://www.larspenke.eu/research/soi-r.html). In addition to a general measure of sociosexual orientation, the inventory also assesses its three facets: behavior, attitude, and desire (3 items per facet). We applied the questionnaire version with 5-point response scales; hence, the result for each facet can assume values from 3 to 15. Participants’ answers were summed up across items with the reverse-coding of Item 6 (Penke & Asendorpf, 2008). Several women declined to provide these data (Table 1).

#### Procedure

Anthropometric measurements were taken by a trained researcher. The examined women were lightly dressed, the type of clothes worn noted in writing, and each woman provided information on the kind of bra she currently wore, if any. Two other women were measured in various clothing and bra type in order to establish their impact on the value of measured circumferences. Then, the appropriate corrections were applied to the values measured. Specifically, we established that, in comparison with a no brassiere state, a bra with soft cups increased the breast circumference, on average, by 1 cm, a bra with rigid cups by 1.5 cm, and a push-up brassiere by 2.5 cm. Breast size was calculated as the difference between breast circumference and chest circumference. We contend that the difference between these circumferences is a more accurate estimation of the breast size than their quotient, which has been used for some previous studies (see the Supplementary Material for details).

To assess the level of asymmetry for each woman, we took color photographs of her face and hands with a digital camera (Panasonic DMC-FZ200, 12.1 MPx). Frontal facial was taken from a distance of 2 m. Subjects were asked to remove their glasses and jewelry, sweep their hair from their face, and display a neutral expression with a direct gaze and lips held gently together. The ventral side of both hands was photographed from a distance of 0.5 m. Participants placed their hands on a white sheet attached to the wall with the dorsal side of the hand flush with the wall, fingers and wrist straightened and in natural arrangement. Subjects were photographed in a standing position and illuminated with the room fluorescent lighting and the camera flash.

To assess estradiol and testosterone levels, saliva samples of 102 women were collected in the mid-luteal phase of their menstrual cycles, which was estimated from the length of the menstrual cycle and the day of last menses as provided by the participant. The mid-luteal phase was chosen because the estradiol concentration is relatively stable during this period, even though for some women there is an estradiol peak at that time (Stricker et al., 2006). We decided not to collect saliva in the fertile (periovulatory) phase because the estradiol level then changes rapidly and determination of the ovulation day-by-day counting method has low accuracy (Blake, Dixson, O’Dean, & Denson, 2016). The date for saliva collection was assigned by the formula: the day of last menses plus the menstrual cycle length minus 7. Participants obtained glass vials one day earlier and were instructed to provide saliva samples by spitting into them the next day shortly after waking up and rinsing the mouth with still water and then to store the vial in a refrigerator. The vial was taken from the participant within several hours and frozen (− 18 °C). Then vials were sent to the laboratory at the Department of Internal Medicine and Endocrinology in Warsaw for assaying.

#### Analysis

To determine repeatability of the breast size assessment, we measured 19 women several months after the main examination. Test–retest correlation was 0.93 for breast circumference, 0.91 for chest girth, and 0.78 for calculated breast size. Differences between the first and second measurements included both measurement error and the actual change in breast size ensuing, for example, from body mass change (Schautz, Later, Heller, Muller, & Bosy-Westphal, 2011).

The number of disease episodes (respiratory or digestive) and their average duration were log-transformed to achieve normal-like distributions. Variables related to respiratory and digestive infections underwent two factorial analyses to obtain indices of respiratory infections and digestive infections, respectively. Factor loadings for the number of diseases, average duration of diseases, and frequency of antibiotic use (the number of disease episodes with antibiotic use divided by the total number of disease episodes) were, respectively, 0.70, 0.77, and 0.64 for respiratory infections and 0.83, 0.92, and 0.63 for digestive infections.

Participants’ photographs were digitally processed. Using Adobe Photoshop software, facial images were rotated to eliminate any head tilting, and a white mask was digitally applied to each photograph so as to hide all extraneous elements around the face (DeBruine, Jones, Smith, & Little, 2010; Little, Jones, & DeBruine, 2011). Photographs were evened out for facial size, which was calculated as the average distance of several landmarks from their centroid: trichion, zygions, gonions, gnathion, pupils, and stomion (Farkas, 1994). We then measured (in pixels) differences between left and right facial side for: (1) eye height, (2) horizontal distance between the mouth corner and face contour, (3) pupil’s y-coordinate, and (4) mouth corner y-coordinate. In addition, the distance of the point lying midway between nasal alae from the line crossing points lying midway between inner eye corners and mouth corners was measured. We thus endeavored to involve measurements that (1) were based on landmarks that can be precisely located on a frontal facial photograph and (2) captured various forms of facial asymmetry, horizontal and vertical, related to shape and to location of facial elements. Values of the five measurements were *z*-scored, which, apart from normalizing the variables, removed possible directional asymmetry from the asymmetry measures, thus preserving only fluctuating asymmetry which is supposedly the only asymmetry type related to biological quality and facial attractiveness (Gangestad & Thornhill, 1999). We then calculated the absolute values for the *z*-scores because we were interested in the magnitude of the fluctuating asymmetry rather than its direction. Finally, the five resultant values for the face were summed to obtain the Index of Facial Asymmetry.

We then digitally rotated the hand images so as to make them visually vertical and measured hand width and length of all digits but thumbs. The length of each finger was taken from its tip to the midpoint of the proximal crease at the digit base (Fink, Manning, Neave, & Grammer, 2004; Manning, 2002). The hand width was taken as the distance between the points where proximal and distal transverse flexure creases reach the hand contour (Kościński, 2012). The original values of the digital measurements were in pixels but were converted into millimeters using a line of length 10 cm that was drawn on the sheet and photographed along with each hand; this ensured comparability between values for the left and right hand. Next, for each of the four digits and for hand width we calculated the relative fluctuating asymmetry: we took the difference between values for the left and right hand, subtracted the mean difference (across individuals) from it (to discard directional asymmetry), calculated the absolute value for the outcome, and divided it by the mean value from the two hands of the individual. In cases of fractures or dislocations of the measured digits (as uncovered in the interview), the calculated asymmetry was substituted with the average relative asymmetry for the given trait in the sample (Hume & Montgomerie, 2001; Thornhill, Gangestad, & Comer, 1995). The resultant values for the five traits were averaged for each subject to obtain the Index of Hand Asymmetry.

Indices of facial and hand asymmetry were log-transformed to achieve normal-like distributions, *z*-scored to even out their variations, and averaged to obtain the Index of Total Asymmetry. To assess the reliability of the asymmetry measurements, we placed all landmarks on photographs of 10 women and calculated the asymmetry indices a second time. The test–retest correlation was 0.91 for Index of Facial Asymmetry and 0.95 for Index of Hand Asymmetry.

Hormone concentrations were estimated with enzyme immunoassay kits from DRG Instruments GmbH (Marburg, Germany), no. SLV-4188 for estradiol and no. SLV-3013 for testosterone. The analysis was conducted for 83 women who declared no use of hormonal contraceptives. Estradiol and testosterone assessments succeeded for 74 and 78 women, respectively (Table 1). The assessments were obtained in duplicate, and the intraassay coefficient of variation was 9.0% for estradiol and 5.9% for testosterone. Distribution of testosterone level was not normal, and we applied log transformation to make it normal-like.

All statistical analyses were conducted using Statistica StatSoft 8.0, and reported *p* values are two-tailed.

### Results

Table 1 shows descriptive statistics for participants’ age, height, weight, body mass index (i.e., the weight in kilograms divided by the square of the height in meters, BMI), breast and chest circumference, breast size, characteristics of respiratory and digestive infections (count, average duration, frequency of antibiotic use), sociosexual orientation and its facets, and estradiol and testosterone levels. It also presents the Pearson correlation between these traits and breast size. It can be seen that breast size was not related to age and stature, but positively correlated with body mass, BMI, and breast circumference. Breast size was unrelated to chest circumference, which is compatible with our previous claim that breast size calculated as the difference between breast and chest girth is not confounded by the chest girth. Conversely, the quotient of breast circumference to chest circumference was negatively correlated with chest girth (*n* = 163, *r* = − .34, *p* < .001) and not related to breast circumference (*n* = 163, *r* = .08, *p* = .298), indicating that the quotient is confounded by the chest girth.

**Fig. 1:**
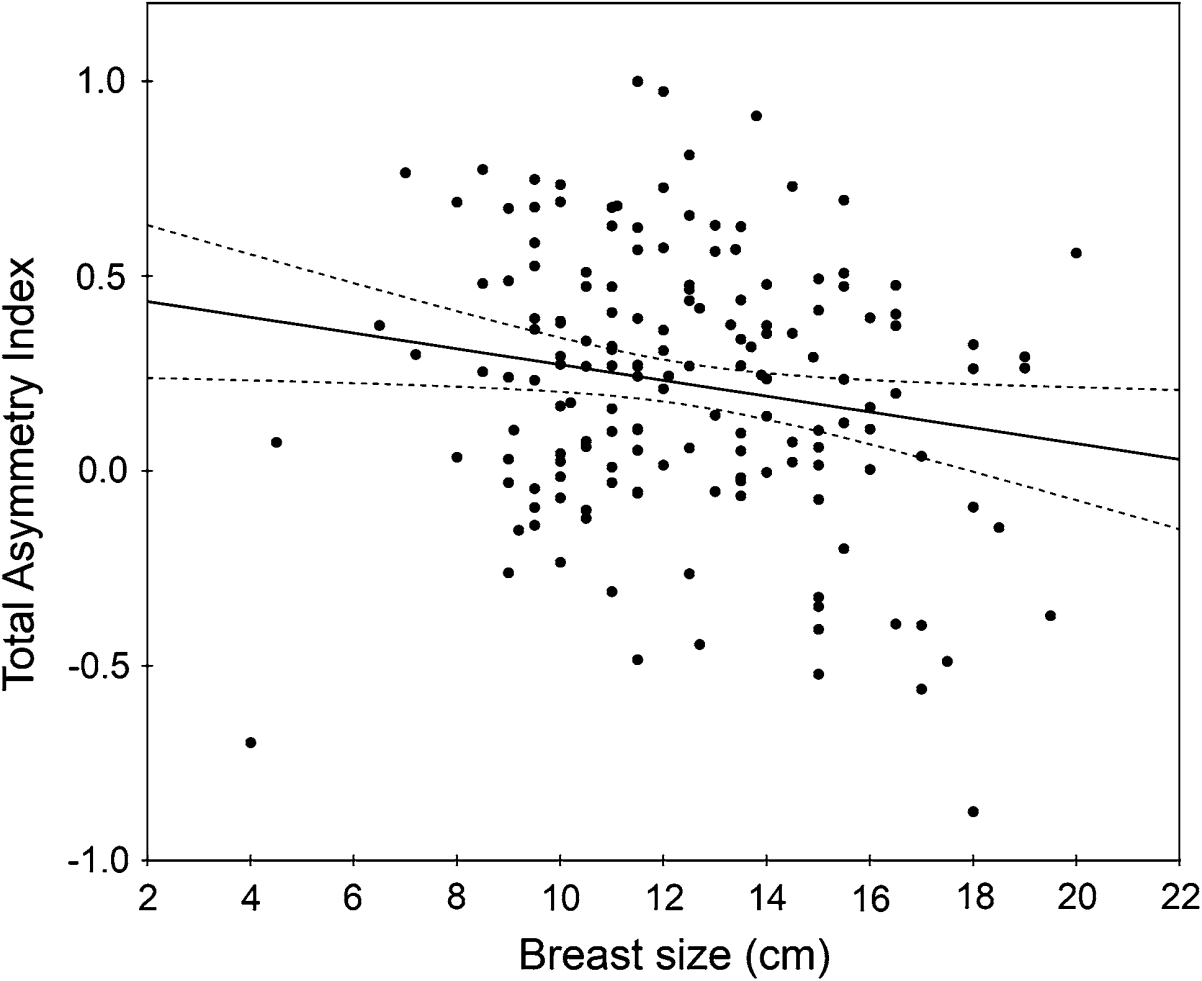
Association between breast size and Total Asymmetry Index (*n* = 163, *r* = − 0.23, *p* = .003). Regression line (solid) and 95% confidence band (intermittent lines) attached to the empirical data (disks)

**Fig. 2:**
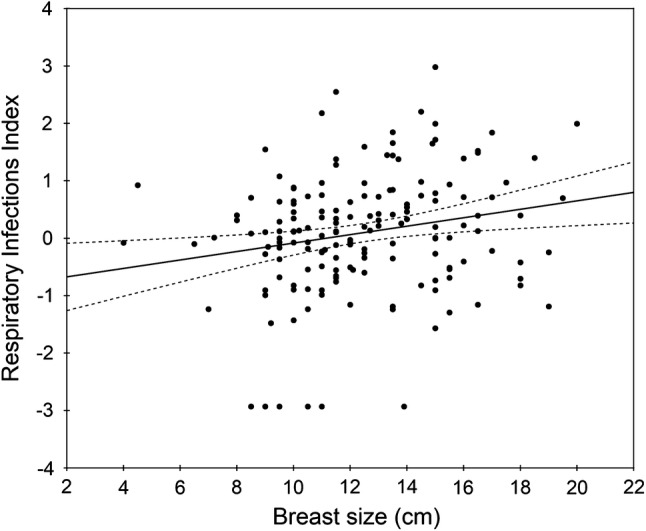
Association between breast size and Respiratory Infections Index (*n* = 163, *r* = 0.21, *p* = .008). Regression line (solid) and 95% confidence band (intermittent lines) attached to the empirical data (disks)

Breast size was negatively associated with Total Asymmetry (Table 1, Fig. 1), indicating that women with larger breasts were in general more symmetric. The correlation coefficient was also negative, though not statistically significant, for each component of Total Asymmetry (Table 1). In addition, breast size was positively related to respiratory infections and two of its components, average duration of illness and frequency of antibiotic use (Table 1, Fig. 2), indicating that women with larger breasts experienced longer episodes of respiratory diseases and took antibiotics more frequently. Breast size was unrelated to estradiol and testosterone levels, digestive infections and each of its components, as well as sociosexual orientation and its components (Table 1). In additional regression analyses, sex hormone levels were also independent of waist-to-hip ratio and interaction between waist-to-hip ratio and breast size (all *p*s > .20).

To exclude the possibility that the relationship between breast size and respiratory infections was produced by the six women who declared no respiratory illness in the past three years and seem somewhat outlying (see Fig. 2), we carried out the respective Pearson correlations for the winsorized respiratory infections where the lowest values were replaced with the minimum value for the remaining participants. Results changed only slightly: *n* = 163, *r* = .20, *p* = .012.

We also wanted to exclude the possibility that relationships of breast size with asymmetry and health history were confounded by body mass. We therefore conducted a series of multiple regression analyses with asymmetry, health or sociosexuality index as the dependent variable and breast size and body mass as two independent variables. These made virtually no impact on the results previously obtained in bivariate analysis: breast size significantly predicted Total Asymmetry (standardized *β* = − 0.16, *p* = .041), respiratory infections (standardized *β* = 0.20, *p* = .011), their duration (standardized *β* = 0.18, *p* = .027), and antibiotic use (standardized *β* = − 0.26, *p* = .001), with still no significant influence on Facial Asymmetry (standardized *β* = − 0.12, *p* = .147), Hand Asymmetry (standardized *β* = − 0.12, *p* = .126), number of respiratory infections (standardized *β* = 0.03, *p* = .738), digestive infections, sociosexual orientation and their components (all *p*s > .3). Very similar results were obtained when the series of regressions was conducted with the possible confounder being body height or an indicator of body shape (body mass index or waist-to-height ratio) instead of body mass.

To test possible curvilinear relationships between breast size and other variables, we conducted a series of multiple regression analyses with Total Asymmetry, respiratory or digestive infections, or sociosexual orientation as the dependent variable and breast size and its square as two independent variables. The squared term was nonsignificant for each case (all *p*s > .07) except for Total Asymmetry (standardized *β* = − 1.31, *p* = .007). However, the last effect lost significance when the outlying point for the woman with breast size of 4 cm (see Fig. 1) was excluded from the analysis (standardized *β* = − 0.73, *p* = .18).

### Discussion

We found that breast size was negatively correlated with body asymmetry and positively with respiratory infections, but unrelated to infections of the digestive system, openness to casual sex, and testosterone and estradiol level. These two significant effects were quite weak, precluding a reliable inference on the health, biological quality, or mate value of an individual woman on the basis of her breast size. However, such findings extend our knowledge on women at the population level and it is known that even weak effects can result in substantial evolutionary changes (e.g., in breast morphology) if operative in the population over many generations (Futuyma, 2009).

We also observed that breast size is positively correlated with body mass. Such relationship has been previously reported in the literature (Brown et al., 2012) and is a result of the association between the amount of adipose tissue in the breast and the body as a whole (Schautz et al., 2011; Wade et al., 2010). In the present study, we conducted analyses that excluded the possibility that relationships between breast size and asymmetry or health history were confounded by body mass.

That bigger breasts are associated with a more symmetric body is compatible with previous studies that found a negative correlation between breast size and relative breast asymmetry (Manning et al., 1997; Møller et al., 1995). It is also compatible with the theory of evolution of biological signals (and the so-called handicap principle), which proposes that traits which are costly to produce and/or maintain should be honest cues to high biological quality of the individual, because only individuals of sufficiently high quality can afford development and maintenance of costly traits (Gangestad & Scheyd, 2005; Zahavi, 1975). A low level of body asymmetry is commonly regarded as a cue to high developmental stability, and thereby high genetic or biological quality (Gangestad & Thornhill, 1999; Van Dongen & Gangestad, 2011), and large breasts can be a costly structure for several reasons. Breasts are composed mainly of fibroglandular tissue (related to milk production and excretion) and adipose tissue (an energy store), while the relative proportion of these tissues varies enormously among women (Lejour, 1997; Vandeweyer & Hertens, 2002). Adipose tissue is energetically dense and its metabolism rate is low; hence, it requires relatively more energy for development and little energy for maintenance; the opposite is true for fibroglandular tissue (Wang et al., 2010; Waterlow, 1981). However, because the total mass of the two breasts is usually about 1 kg (Cox, Kent, Casey, Owens, & Hartmann, 1999; Jansen et al., 2014), the above-mentioned energetic costs, although not negligible, cannot be burdensome for an organism. Breasts are also mechanically costly due to their weight and unsteadiness, which raise various forms of pain, especially during body movements (Kerrigan et al., 2001; Spector & Karp, 2007). This must have been particularly important in the evolutionary past when women wore no bras. Another form of cost is related to breasts being an element of inter-female rivalry (Fink, Klappauf, Brewer, & Shackelford, 2014). Intrasexual rivalry can be costly, particularly for individuals who unreliably signal their high quality (Berglund, Bisazza, & Pilastro, 1996; Mueller & Mazur, 1997). One more cost is related to breast sagging, which is unattractive to men (Groyecka, Zelaźniewicz, Misiak, Karwowski, & Sorokowski, 2017; Havlíček et al., 2017), and larger breasts are more likely to sag (Rinker, Veneracion, & Walsh, 2008; Soltanian, Liu, Cash, & Iglesias, 2012). Although the fitness consequences of the above-mentioned costs and problems related to development and maintenance of big breasts have not been measured, they are arguably not negligible. It is possible that these costs are lower for women characterized by relatively high biological quality.

The observed positive correlation between breast size and respiratory infections may seem to contradict the claim that size of costly traits (e.g., breasts) should be positively associated with the individual’s quality, including health. However, evolutionary biology theorists have argued that it sometimes pays individuals of higher genetic quality to invest in mating at the expense of health to the extent that they are more attractive and have higher reproductive success but are less healthy than conspecifics of a lower quality (Getty, 2002; Kokko, 2001). Indeed, studies on nonhuman animals revealed both positive and negative correlations between putative cues to genetic quality and survival rate (Jennions, Møller, & Petrie, 2001). It is therefore possible that women of high biological quality have more symmetric bodies (because of higher developmental stability), larger breasts (because they can afford it), and worse infectious health (because of a particular life strategy being adopted).

The negative relationships of breast size with infectious health obtained here are also compatible with literature findings that large breasts are associated with higher risk of type 2 diabetes (Ray et al., 2008) and breast cancer (Jansen et al., 2014). The risk of breast cancer is supposedly increased both by amount of fibroglandular tissue, via the number of epithelial cells which can become cancerous, and adipose tissue, due to its carcinogenous properties (Boyd et al., 2007; Jansen et al., 2014). Hormonal activity of adipose tissue in breasts has been suggested to be responsible for the influence of breast size on the risk of diabetes (Ray et al., 2008). Since excessive adipose tissue in body is associated with decreased function of the immune system (Rantala et al., 2013; Samartín & Chandra, 2001), it is possible that the correlation between breast size and respiratory infections observed here was mediated by the amount of adipose tissue in the breasts.

We found that breast size was associated with length of respiratory infections and frequency of antibiotic use but not with number of respiratory infections and any parameter describing digestive infections. Correlations with respiratory but not digestive infections have been already reported for facial appearance (Gray & Boothroyd, 2012; Thornhill & Gangestad, 2006). The individual’s immunocompetence is arguably better reflected in the length of illness episodes (the time the organism needs to eliminate pathogens) and frequency of antibiotic use (which is associated with severity of diseases) than the number of episodes, which depends, to a higher degree, on exposure to pathogens. The reason for lack of significant correlations for digestive infections may be that they are much less frequent than respiratory ones.

It should be also noted that we relied on health data as declared by the participants. Such data are commonly used in epidemiological studies because they are easy to collect at low cost and in a short time. Although some researchers pointed to limited accuracy of the method (Savilahti, Uitti, & Husman, 2005), others defended its usability (Stevenson, Case, & Oaten, 2009). If the error in providing health data was associated with breast size, a spurious correlation between infectious health and breast size could be revealed. Our results require replication with an objective measure of infectious health, e.g., the response of the immune system by antibody production (Rantala et al., 2013).

We found no significant relationship between breast size and sex hormone levels. This corresponds with results reported by Garver-Apgar et al. (2011) and Grillot et al. (2014) but not with Jasieńska et al. (2004), who found that women with larger breasts have a higher level of estradiol. It is possible that our sample was too small to demonstrate a significant association for such a labile trait as concentration of estradiol or testosterone (Shultz, Wideman, Montgomery, & Levine, 2011; Stricker et al., 2006), all the more so that we had only one saliva sample per woman. Alternatively, a biologically meaningful correlation between breast size and sex hormone levels may not exist. Jasieńska et al. estimated breast size with the breast-to-chest circumference ratio, which is severely confounded with chest circumference (see the Supplementary Material). Their results may therefore pertain to chest size rather than breast size. Further research is needed to clarify this.

Breast size proved unrelated to a woman’s sociosexual orientation and each of its facets. Therefore, no support was found for Smith’s (1984) hypothesis that ancestral women evolved permanent breasts in order to facilitate cheating of the partner. This hypothesis has previously been criticized on theoretical grounds (Caro, 1987; Marlowe, 1998), and our study failed to provide any empirical support for it.

## Study 2

### Method

#### Participants

The study involved two groups of participants. The first group participated in Study 2A and included 140 women and 125 men, aged 18–58 years (*M* = 23.2, SD = 4.1). The second group participated in Study 2B and included 154 women and 98 men, aged 18–59 years (*M* = 26.1, SD = 6.6). Participants were recruited opportunistically among acquaintances and students of universities in Poznań, all being of European descent. They were each given a web page address and asked to complete the questionnaire therein.

#### Measures

To determine the appearance of average female breasts, we identified and photographed a woman (aged 33, Caucasian origin) who had average breast size and other morphological characteristics (see Table 1): height = 167 cm, weight = 60 kg, BMI = 21.5 kg/m^2^, breast girth = 86 cm, chest girth = 73 cm, breast size = 13 cm (86–73). The woman was photographed from the front, standing, and topless. The photograph was taken from a distance of 2 m, and the frame encompassed the body from hips to neck. Digital female figures were manufactured with MakeHuman 1.0.2, freeware software designed to model three-dimensional characters (http://www.makehuman.org). Images of the virtual and the real woman were zoomed to the same size and placed side by side on the screen. Breast size in the virtual woman was modified with the breast size slider so as to achieve an appearance compatible with that on the photograph of the real woman.

Next, we endeavored to set the model’s breast size to be 1 SD below or above the average size. We assumed a SD of breast size as 3 cm (see Table 1) and the average width of the chest at the breast level as 28 cm (measured on the photographed woman with a spreading caliper). Making use of the fact that breast circumference can be approximated with a sum of appropriate segments and arcs (see the Supplementary Material), we calculated that a decrease or increase in the radius of the sphere for each breast by about 1.0 cm or 3.6% of the chest width would, respectively, decrease or increase the breast circumference by 1 SD (due to change in the breast width and protrusion). Such changes were therefore applied to the breasts of the virtual female model.

In order to lessen explicit use of stereotypes related to breast size, we intended not to focus participants’ attention specifically to the breasts. For this reason, people were invited to participate in a study on the “female body” rather than “breasts” and the figures presented to them differed not only in breast size but also in hip size (by ± 10%) and hair color (black, dark blond, and red). Variation in hip size and hair color is easily perceptible and related to attractiveness and social perception (Janif, Brooks, & Dixson, 2014; Rozmus-Wrzesińska & Pawłowski, 2005; Swami et al., 2008). Hip width was modified by the technique of warping with author-developed software (Kościński, 2013, 2014), and hair color was changed with Adobe Photoshop application. Altogether, 27 versions of the female figure were manufactured: 3 breast sizes × 3 hip widths × 3 hair colors (see Fig. S1 in the Supplementary Material for example images).

#### Procedure

Female figures were assessed via an internet page by invited persons (acquaintances, students of local schools, participants from Study 1). Each judge was associated with a random triplet of figures (one of 36 possible triplets) which differed from one another in breast size, hip width, and hair color. These three silhouettes were displayed on the screen next to each other in random order, and an inscription asked the judge to assess each woman on a 1–7-point scale in regard to a specific characteristic. Above each female image were placed 7 option boxes labeled from 1 to 7. After making the assessments, the participant clicked the “Ready” button to proceed to evaluate the same female images for another characteristic. The order of judged characteristics was constant across all participants.

The first group of participants (in Study 2A) judged 5 traits: (1) physical attractiveness, from 1 labeled as “the lowest attractiveness” to 7 labeled as “the highest attractiveness,” (2) sexual desire, 1 “the weakest sexual desire” to 7 “the strongest sexual desire,” (3) sexual promiscuity, 1 “unwilling to engage in casual sex” to 7 “willing to engage in casual sex,” (4) reproductive efficiency, 1 “has problems with getting pregnant and labor” to 7 “gets pregnant and gives births with no problem,” (5) lactational efficiency, 1 “low quantity and quality of breast milk” to 7 “high quantity and quality of breast milk.”

The second group of participants (in Study 2B) judged the other 5 traits: (1) marital attractiveness, 1 “inappropriate for a wife” to 7 “appropriate for a wife,” (2) sexual attractiveness, 1 “inappropriate for a lover” to 7 “appropriate for a lover,” (3) fidelity, 1 “unfaithful to her partner” to 7 “faithful to her partner,” (4) intelligence, 1 “the lowest intelligence” to 7 “the highest intelligence,” (5) diligence, 1 “very lazy” to 7 “very diligent.”

### Results

Preliminary analyses showed that judge age and the location of a silhouette on the screen did not impact on the evaluations obtained and were thus not included in subsequent analyses.^1^ We then tested whether judge sex affected breast evaluations. Two GLM analyses were carried out with five dependent variables (the evaluated traits), three independent variables (judge identity, breast size, and sex, with identity being a random factor nested in sex), and interaction between sex and breast size. Judge sex proved to be a significant predictor [Study 2A: Wilks’ *λ* = 0.91, *F*(5, 522) = 10.70, *p* < .001, Study 2B: Wilks’ *λ* = 0.88, *F*(5, 496) = 13.97, *p* < .001] though its interaction with breast size was not [Study 2A: Wilks’ *λ* = 0.99, *F*(10, 1044) = 0.57, *p* = .84, Study 2B: Wilks’ *λ* = 0.99, *F*(10, 992) = 0.46, *p* = .92]. The sex of judges was therefore included in subsequent analyses.

**Fig. 3:**
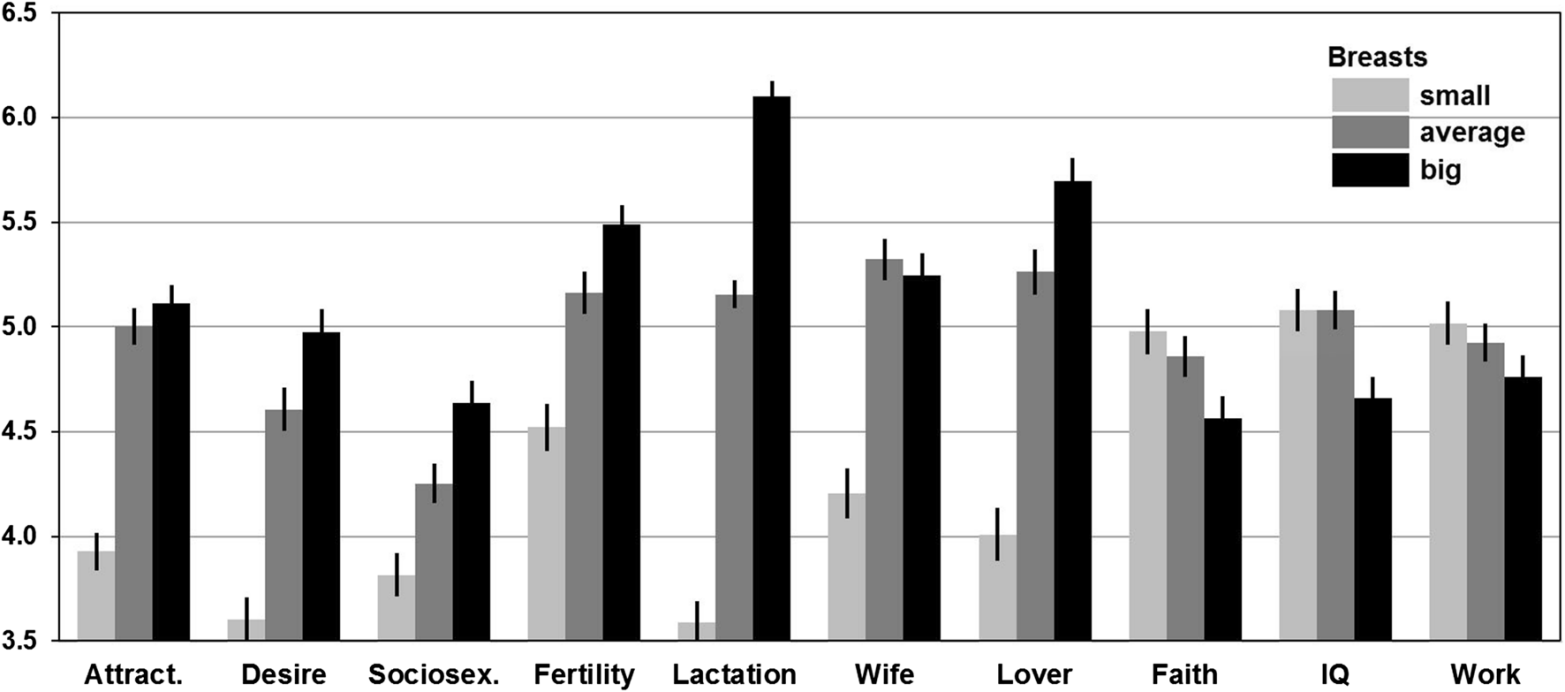
Means (± standard errors) of evaluated traits in relation to the woman’s breast size. Labels: Attract.—physical attractiveness, Desire—sexual desire, Sociosex.—sociosexual orientation, Fertility—reproductive efficiency, Lactation—lactational efficiency, Wife—marital attractiveness, Lover—sexual attractiveness, Faith—faithfulness, IQ—intelligence, Work—diligence

Further, we conducted a series of GLM analyses with one dependent variable (an evaluated trait), five independent variables (judge identity, sex, breast size, hip width, and hair color, with identity being a random factor nested in sex), and three interactions: breast size × sex, breast size × hip width, and breast size × hair color. Table 2 shows the results of these analyses, Fig. 3 shows the means of the evaluated traits in relation to breast size, and Table S1 (in Supplementary Material) provides the numerical values of the means for variants of breast size, hip width, and hair color as well as the significance of the differences between the means according to the Tukey test.

**Table 2:** Evaluation of 10 characteristics in digital female figures in relation to breast size, hip size, hair color, and judge sex (results of GLM analyses including partial eta-squared, $\eta_{\text{p}}^{2}$)

	SS	*df*	MS	*F*	*p*	$\eta_{\text{p}}^{2}$
Physical attractiveness						
Intercept	16,881.09	1	16,881.09	8602.30	< .001	0.97
Identity	516.36	263	1.96	1.06	.284	0.35
Sex (S)	0.18	1	0.18	0.09	.762	0.00
Breast size (B)	230.50	2	115.25	62.32	< .001	0.20
Hip width (H)	113.95	2	56.98	30.81	< .001	0.11
Hair color (C)	3.40	2	1.70	0.92	.400	0.00
B × S	4.50	2	2.25	1.22	.297	0.00
B × H	8.04	4	2.01	1.09	.362	0.01
B × C	11.36	4	2.84	1.54	.191	0.01
Error	950.57	514	1.85			
Sexual desire						
Intercept	15,002.69	1	15,002.69	3007.57	< .001	0.92
Identity	1319.11	263	5.02	2.88	< .001	0.60
Sex (S)	59.82	1	59.82	11.97	.001	0.04
Breast size (B)	262.10	2	131.05	75.29	< .001	0.23
Hip width (H)	59.48	2	29.74	17.09	< .001	0.06
Hair color (C)	2.60	2	1.30	0.75	.474	0.00
B × S	0.23	2	0.11	0.07	.937	0.00
B × H	9.32	4	2.33	1.34	.254	0.01
B × C	6.69	4	1.67	0.96	.429	0.01
Error	894.63	514	1.74			
Sociosexual orientation						
Intercept	13,812.55	1	13,812.55	3195.49	< .001	0.92
Identity	1142.46	263	4.34	2.45	< .001	0.56
Sex (S)	0.06	1	0.06	0.01	.909	0.00
Breast size (B)	79.45	2	39.72	22.39	< .001	0.08
Hip width (H)	2.39	2	1.20	0.67	.510	0.00
Hair color (C)	22.40	2	11.20	6.31	.002	0.02
B × S	1.50	2	0.75	0.42	.656	0.00
B × H	5.16	4	1.29	0.73	.574	0.01
B × C	15.60	4	3.90	2.20	.068	0.02
Error	911.78	514	1.77			
Reproductive efficiency						
Intercept	19,804.04	1	19,804.04	8083.15	< .001	0.97
Identity	645.91	263	2.46	1.40	.001	0.42
Sex (S)	9.08	1	9.08	3.70	.055	0.01
Breast size (B)	89.70	2	44.85	25.63	< .001	0.09
Hip width (H)	612.01	2	306.00	174.87	< .001	0.40
Hair color (C)	0.05	2	0.02	0.01	.987	0.00
B × S	0.14	2	0.07	0.04	.961	0.00
B × H	3.94	4	0.98	0.56	.690	0.00
B × C	2.85	4	0.71	0.41	.803	0.00
Error	899.47	514	1.75			
Lactational efficiency						
Intercept	18,940.36	1	18,940.36	6900.07	< .001	0.96
Identity	725.17	263	2.76	2.16	< .001	0.52
Sex (S)	5.65	1	5.65	2.06	.153	0.01
Breast size (B)	808.68	2	404.34	316.51	< .001	0.55
Hip width (H)	7.83	2	3.92	3.07	.047	0.01
Hair color (C)	1.56	2	0.78	0.61	.544	0.00
B × S	1.81	2	0.91	0.71	.492	0.00
B × H	5.13	4	1.28	1.00	.405	0.01
B × C	4.82	4	1.20	0.94	.439	0.01
Error	656.63	514	1.28			
Marital attractiveness						
Intercept	16,877.92	1	16,877.92	4531.83	< .001	0.95
Identity	930.73	250	3.72	1.56	< .001	0.44
Sex (S)	32.28	1	32.28	8.67	.004	0.03
Breast size (B)	184.00	2	92.00	38.63	< .001	0.14
Hip width (H)	53.54	2	26.77	11.24	< .001	0.04
Hair color (C)	0.97	2	0.49	0.20	.815	0.00
B × S	3.87	2	1.94	0.81	.444	0.00
B × H	4.32	4	1.08	0.45	.770	0.00
B × C	4.19	4	1.05	0.44	.780	0.00
Error	1162.32	488	2.38			
Sexual attractiveness						
Intercept	17,528.63	1	17,528.63	3980.45	< .001	0.94
Identity	1100.45	250	4.40	1.67	< .001	0.46
Sex (S)	4.66	1	4.66	1.06	.305	0.00
Breast size (B)	366.05	2	183.02	69.61	< .001	0.22
Hip width (H)	71.80	2	35.90	13.65	< .001	0.05
Hair color (C)	3.46	2	1.73	0.66	.518	0.00
B × S	4.64	2	2.32	0.88	.415	0.00
B × H	10.87	4	2.72	1.03	.389	0.01
B × C	9.00	4	2.25	0.86	.491	0.01
Error	1283.12	488	2.63			
Faithfulness						
Intercept	15,898.25	1	15,898.25	3848.21	< .001	0.94
Identity	1032.21	250	4.13	2.35	< .001	0.55
Sex (S)	37.71	1	37.71	9.14	.003	0.04
Breast size (B)	18.93	2	9.47	5.38	.005	0.02
Hip width (H)	15.02	2	7.51	4.27	.015	0.02
Hair color (C)	4.67	2	2.34	1.33	.266	0.01
B × S	1.67	2	0.84	0.47	.622	0.00
B × H	13.78	4	3.44	1.96	.100	0.02
B × C	18.17	4	4.54	2.58	.037	0.02
Error	859.08	488	1.76			
Intelligence						
Intercept	16,796.59	1	16,796.59	3749.60	< .001	0.94
Identity	1119.06	250	4.48	3.49	< .001	0.64
Sex (S)	71.51	1	71.51	15.99	< .001	0.06
Breast size (B)	28.26	2	14.13	11.03	< .001	0.04
Hip width (H)	6.33	2	3.16	2.47	.086	0.01
Hair color (C)	1.67	2	0.83	0.65	.522	0.00
B × S	0.91	2	0.46	0.36	.700	0.00
B × H	6.79	4	1.70	1.32	.260	0.01
B × C	4.17	4	1.04	0.81	.517	0.01
Error	625.29	488	1.28			
Diligence						
Intercept	16,667.05	1	16,667.05	3842.20	< .001	0.94
Identity	1083.71	250	4.33	3.05	< .001	0.61
Sex (S)	31.14	1	31.14	7.19	.008	0.03
Breast size (B)	7.73	2	3.87	2.72	.067	0.01
Hip width (H)	0.98	2	0.49	0.34	.709	0.00
Hair color (C)	0.01	2	0.00	0.00	.997	0.00
B × S	0.13	2	0.06	0.04	.957	0.00
B × H	17.59	4	4.40	3.09	.016	0.02
B × C	7.05	4	1.76	1.24	.293	0.01
Error	694.26	488	1.42

As can be seen from Table 2, breast size influenced all evaluated traits except diligence. Physical attractiveness did not differ significantly between big and average breast conditions, although small breasts were clearly the least attractive. The same pattern was observed for marital attractiveness. Perception of sexual attractiveness was similar, but big-breasted women received significantly higher evaluations than those with average breasts. Perceived sexual desire, openness to casual sex, reproductive efficiency, and lactational efficiency all increased along with breast size, and the effect was particularly strong for the last trait (see differences between means and the value of partial *η*^2^). Big-breasted women were perceived as less faithful and less intelligent than women with average or small breasts, while the latter two did not differ from each other. Two interactions were observed (Table 2): one interaction indicated that attribution of infidelity to large-breasted women occurred only for the condition of black hair. The other suggested that, among slim women, large breasts were associated with low diligence.

Although effects of hip width and hair color were beyond our scientific interest, we note that some of them were significant (Tables 2 and S1). Women with average hips obtained highest ratings for physical, marital, and sexual attractiveness, and perceived sexual desire. Along with hip width, there was a substantial increase in perceived reproductive efficiency and a slight increase in lactational efficiency. Woman with wide hips were perceived as more faithful than the others. Perceived openness to causal sex, intelligence, and diligence were independent of hip width. There was only one main effect of hair color: red-haired women were perceived to be more open to casual sex than other women (Tables 2 and S1).

There were several effects of judge sex: men, in comparison to women, attributed to the female figures higher sexual desire and lower marital attractiveness, faithfulness, intelligence, and diligence. Judge identity was a significant factor for judgments of each trait except physical attractiveness (Table 2), which simply means that some participants tended to give higher ratings than others.

### Discussion

In agreement with Dixson et al. (2015), we found that women and men were similar to each other in assessments of female figures. Even though several main effects of sex were observed, there was no significant interaction between sex and breast size, which indicates similarity in the pattern of breast size evaluations.

Physical attractiveness and marital attractiveness of large breasts were very similar to attractiveness scores for average breasts, while the attractiveness of a small bust was decisively lower. Evaluation of sexual attractiveness was similar, except that women with large breasts received somewhat higher ratings than those with average breasts. This difference between marital (and physical) and sexual attractiveness can be easily explained by the judges perceiving large-breasted women as more promiscuous, less faithful, and less intelligent, and these characteristics being known to be much more preferable or acceptable in the context of a short-term than a long-term relationship (Buss & Schmitt, 1993; Regan, Levin, Sprecher, Christopher, & Cate, 2000). In general, our results for perception of breast attractiveness are compatible with those reported in studies that applied visual stimuli not calibrated for the breast size distribution in the local population (Dixson et al., 2011b, 2015; Gründl et al., 2009; Swami & Tovée, 2013a, b; Zelazniewicz & Pawlowski, 2011).

Similar judgments of marital (and physical) attractiveness for large and average breast versions can be a result of the perception that large-breasted women, in comparison with those with average breasts, possess both desirable (reproductive efficiency, lactational efficiency, sex drive, maybe openness to casual sex) and undesirable characteristics (low faithfulness, low intelligence). We are unable to determine whether these two weak negative attributes in big-breasted women can counterbalance the four positive attributes, including one that is very strong (lactational efficiency). If one considers this as being possible, the observed preference pattern would appear as “rational” (i.e., derived from the attributes). Alternatively, if one finds such counterbalance unlikely, the preferences would be “irrational” (i.e., incompatible with the attributes). The reason for preference “irrationality” may be an automatic process in attractiveness perception that could be an evolutionary adaptation with a genetic foundation or an effect of perceptual biases which results from the manner in which the neural system works.

If perception of breast attractiveness is an evolutionary adaptation, one would expect that the preference pattern is congruent with dependence of female biological quality on breast size. However, previous studies and the present Study 1 showed that the association between breast size and the woman’s quality is complex (e.g., large breasts cue high developmental stability and poor physical health), which makes the dependence of biological fitness on breast size difficult to determine. Small breasts proved much less attractive than the average or large ones and we can only speculate whether they cue a lowered fitness. Although previous human studies suggest that milk production is independent of breast size, it is clear that some minimal amount of mammary glandular tissue is required to effectively nurse the infant (Linzell, 1972). Insufficient development of the mammary gland prevents successful breastfeeding and affects approximately 5% of women (Neifert, 2001). This condition is strongly associated with tuberous breast syndrome (Huggins, Petok, & Mireles, 2000), which is frequently characterized by a small breast volume (von Heimburg, Exner, Kruft, & Lemperle, 1996). Rinker et al. (2008) found that women with small breasts before their first pregnancy (cup size A) breastfed their children more than two times less frequently than those with breasts of size B, C, or D. It is possible that some of the A-size women were affected with a glandular hypoplasia.

Interestingly, the observed pattern of preference for breast size can also be explained by perceptual biases. The neural system processes objects that are typical for their category more efficiently than atypical ones, which makes typical objects of any category regarded as being more attractive than others (Halberstadt, 2006). Another bias is related to discrimination between categories: if objects from one category are preferred to objects from the other (e.g., one sex over the other in mating context), then objects with exaggerated features specific for the preferred category will be particularly attractive because they are discriminated more easily by the neural system (Ghirlanda, Jansson, & Enquist, 2002). The first bias would lead to a preference for typical or average breasts over small and large, while the second one would lead to the “the bigger—the more attractive” pattern. The joint effect of both can produce the pattern of attractiveness we observed in the present study: large and average breasts of similar attractiveness with small breasts being minimally attractive.

One further mechanism that could be responsible for the observed pattern of preference for breast size relates to breast appearance providing cues to the woman’s biological age. Breasts become more and more saggy with age and subsequent pregnancies (Arefanian et al., 2018; Rinker et al., 2008; Soltanian et al., 2012). Breast sagging is therefore a cue to biological condition and, thereby, the potential to reproduce in future (Jasienska, 2009). Breast shape may then be, and might have been in human ancestors, used by men to assess women’s reproductive potential. Because larger breasts are more likely to sag (Arefanian et al., 2018; Rinker et al., 2008; Soltanian et al., 2012), the possibility of inferring a woman’s biological age from the breast shape is limited for small breasts. This may be a reason of relatively low attractiveness of small breasts (Marlowe, 1998).

Our participants, as those in the study by Furnham et al. (1998), thought that breast size is positively correlated with the woman’s openness to casual sex but Study 1 did not find such a correlation. In accordance with this assumed confidence, men solicit or help foreign women more frequently if they have large breasts (Guéguen, 2007a, b; Lynn, 2009). Such erroneous confidence leads therefore to behavior that is suboptimal in terms of mating efficiency (and thereby evolutionarily maladaptive), and also socially harmful if it makes large-breasted women feel uncomfortable. This stereotype may have developed due to erotic and pornographic media which promote models with above-average breasts (Tovée, Mason, Emery, McCluskey, & Cohen-Tovée, 1997; Voracek & Fisher, 2006).

Another example of stereotypical perception of the female bust is the strong association between size of breasts and lactational efficiency attributed to them. As noted above, actual lactational efficiency is at most weakly correlated with breast size. Statistically, women with average breasts have the same capacity to breastfeed successfully as large-breasted females, and this capacity may be lowered only in women having small breasts. However, people perceive the potential for milk production to be much higher in women with a larger than average bust. This pattern of judgments seems to be an example of a stereotypical reasoning “the bigger appliance–the higher capacity.” Participants also associated breast size with reproductive efficiency, but there exists little empirical evidence that breast size is correlated with high estradiol or low testosterone level, i.e., hormonal profiles characteristic for women of a good reproductive health (see the Discussion in Study 1).

### Conclusions

In the present studies, we aimed to extend knowledge on the relationships between breast size and some mating-relevant characteristics and on the perception of women in relation to their breast size. The size of breasts proved to be positively associated with body symmetry, and thereby developmental stability which cues to high biological quality, and negatively with infectious health. This suggests that the relationship between breast size and biological quality or mate value is complex and one cannot infer that women of some breast size are biologically “better” than others. However, it is also possible that large breasts indeed signal higher genetic quality if women with good genes invest in mating (among others, large breasts) at the expense of health. It remains to be established whether the correlation between breast size and health results from a causal relationship between them or is an effect of interindividual variation in energy allocation for mating versus the immune system.

In line with previous research conducted on non-calibrated stimuli, we found that large-breasted women were of similar attractiveness to women with average breasts and much more attractive than females with a small bust. This pattern of preference can be reasonably explained in several ways, with reference to (1) evolutionary adaptation acting via mental automatism, (2) rational analysis of breast-related stereotypes, (3) perceptual biases, or (4) signaling of reproductive potential via breast firmness. We suggest that future studies investigating interindividual variation in preferences and stereotypes pertaining to breasts can further elucidate this issue. We also uncovered that attribution of sexual permissiveness to large-breasted women is an incorrect stereotype since openness to casual sex proved to be unrelated to breast size. Men who seek short-term relationships should be aware that a large bust is not a cue to a woman’s promiscuity. There also exist strong stereotypes associating breast size with reproductive and, particularly, lactational efficiency even though any real relationships between these traits are uncertain.

We recognize that our studies were carried out in one of the so-called WEIRD (Western, educated, industrialized, rich, and democratic) societies, where breast perception and signals may differ from those in traditional cultures or our ancestors: they require replication in more traditional societies. Future research should also investigate the dependence of breast perception and preference on men’s characteristics such as mate value, accessibility to partners, erotic experience, family background.

## Electronic supplementary material

Below is the link to the electronic supplementary material.

Supplementary material 1 (DOC 646 kb)

## Footnotes

1 The statistical results are available from the corresponding author upon request.
