# Supplementary material for: Stereotypical and Actual Associations of Breast Size with Mating-Relevant Traits
Source: Arch Sex Behav. 2019 Sep 27;49(3):821–36. doi: 10.1007/s10508-019-1464-z (PMC7058577; doi:10.1007/s10508-019-1464-z)
Supplement: Supplementary file 1 — Supplementary material 1 (DOC 646 kb) [file 10508_2019_1464_MOESM1_ESM.doc]

**Supplementary Material to the paper “Stereotypical and actual associations of breast size with mating-relevant traits”**

*Breast-Chest Girth Difference vs. Breast-to-Chest Girth Ratio as estimators of breast size*

Various methods have previously been applied to estimate breast size in women. Some of them are highly accurate but expensive, time-consuming or potentially invasive of the intimacy of the woman; these include magnetic resonance imaging, mammography, use of plastic cones with a scale, water displacement procedure, casting, 3D scanning, and anthropometric measurements on naked breasts (Kayar et al. 2011). To circumvent these inconveniences, researchers frequently measure just two body girths: at the level of breasts (breast girth) and just below the breasts (chest girth). However, this method is obviously afflicted with some inaccuracy in that breast circumference depends only on the sagittal (anterior-posterior) and transverse, but not the vertical, breast dimensions. Furthermore, a question arises on how the data for breast and chest circumference should be combined to obtain the best estimation of breast size. Some studies calculated the difference between them (e.g., Brown et al. 2012). Many studies relied on the brassiere cup size, which derives from the difference too (e.g., Lynn 2009, Ray et al. 2008, Jansen et al. 2014). Other studies, however, used the quotient of breast to chest girth (e.g., Jasieńska et al. 2004, Garver-Apgar et al. 2011, Grillot et al. 2014)[[1]](#footnote-2).

To determine which method provides a better estimation of breast size, we conducted two models: the first was material and involved models of chest and breasts made of salt dough, while the second was digital and relied on ellipses and circles of varying size depicting horizontal cross-sections of the chest and breast, respectively.

1. Material model. We used salt dough to make two ellipses, small and large, depicting the horizontal section of two trunks (small and large) at the breast level, and two pairs of moon-shaped elements depicting horizontal sections of small and large breasts (Fig. 1). Next, we attached breast pairs to chests in four ways (small/big chest × small/big breasts, Fig. 2) and measured breast girth with a measuring tape (Fig. 3). In addition, the circumference of the elliptic elements (“chests”) alone were measured as an estimate of chest girth just below the breasts. Then the breast-chest difference and breast-to-chest quotient were calculated for each of four female models. The results are illustrated on Figs. 4-5. It is clear that breast-chest difference depends mainly on the breast size and minimally on the chest size (Fig. 4), whereas breast-to-chest ratio is severely confounded with chest size: two women with identical breasts have very different values of this ratio if their trunks are of different sizes (Fig. 5). The difference between breast girth and under-breast girth seems therefore to be a satisfactory estimator of the breast size but the ratio of these two girths does not.

2. Digital model. The horizontal section of the body at the level of breasts was modeled with an ellipse depicting the chest and two circles depicting breasts (Fig. 6). Chest girth was estimated as the ellipse perimeter. Breast girth was calculated as the sum of several segments and arcs which outline the route of a measuring tape during measurement of breast girth (Fig. 6). Breast size and chest size each varied from 2.5 standard deviations (SD) below the mean of the respective trait to 2.5 SD above the mean[[2]](#footnote-3). Each time, the breast-chest difference and breast-to-chest quotient were calculated. Figure 7 presents the dependence of breast-chest difference on breast size for several variants of chest size. Figure 8 presents similar data for breast-to-chest ratio. As for the material model, it is clear that the breast-chest difference depends almost exclusively on the breast size and therefore is a satisfactory estimator thereof (Fig. 7). Conversely, breast-to-chest ratio depends strongly on both breast and chest size (Fig. 8) and therefore cannot be regarded a reliable estimator of the breast size.

Conclusion: Both models clearly demonstrated that the breast-chest girth difference is a much better estimator of breast size than the breast-to-chest girth ratio.


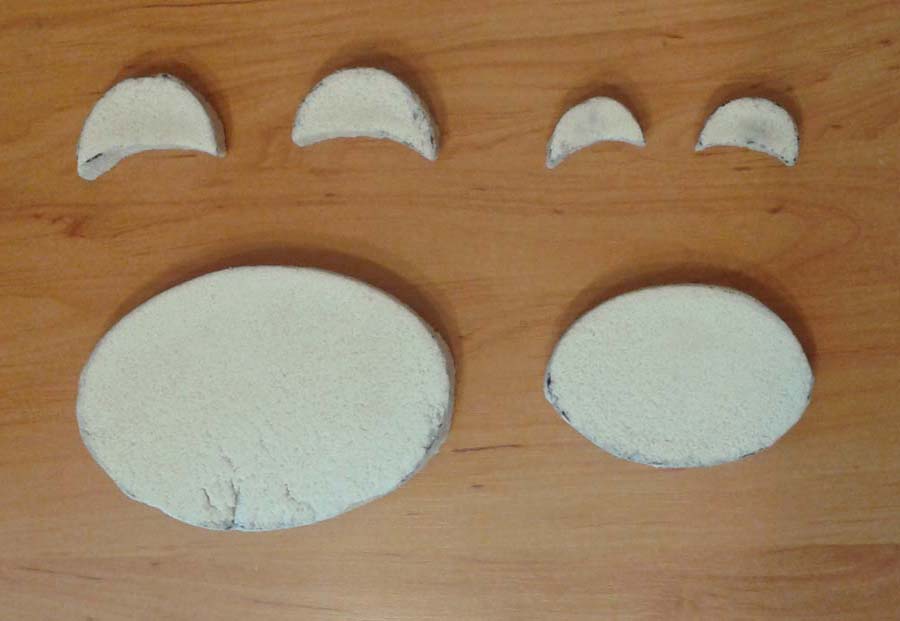


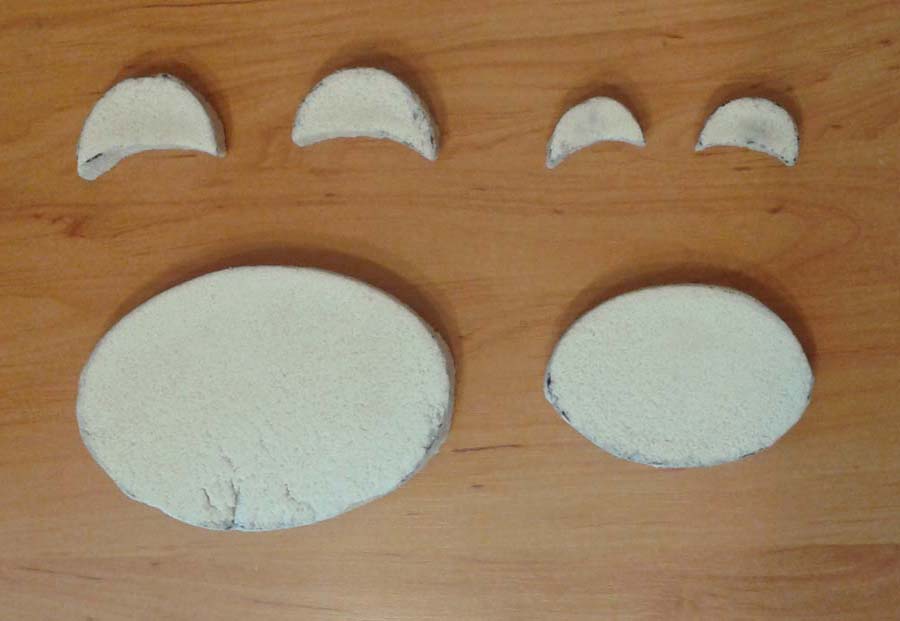
Fig. 1. Salt dough models for horizontal sections of big and small chest (elliptic elements) and breasts (moon-shaped elements).


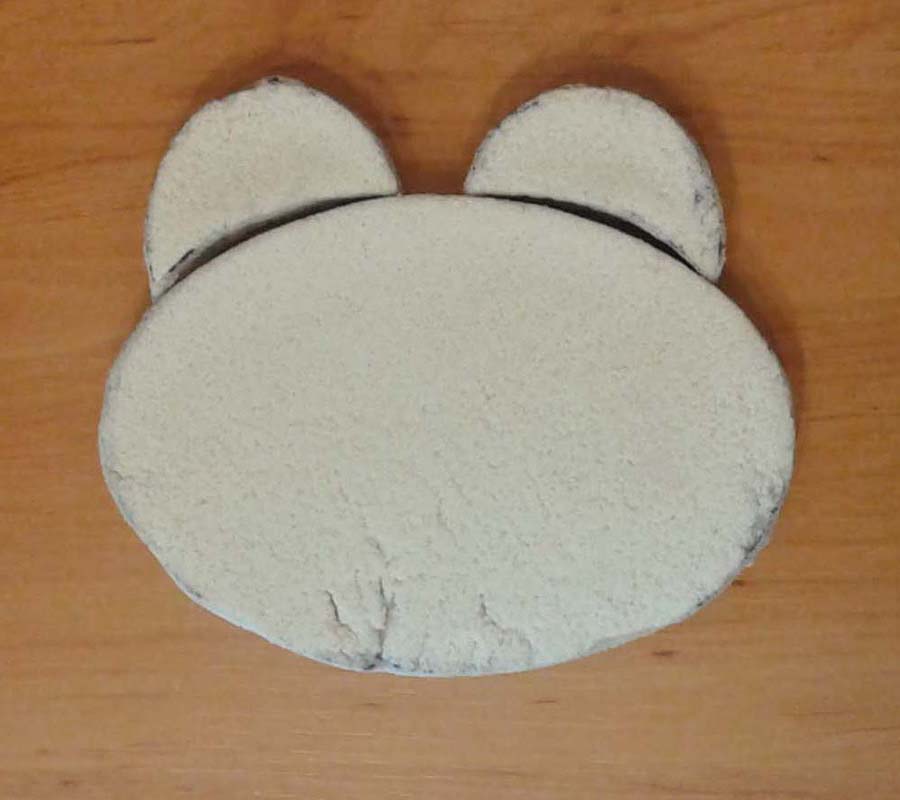

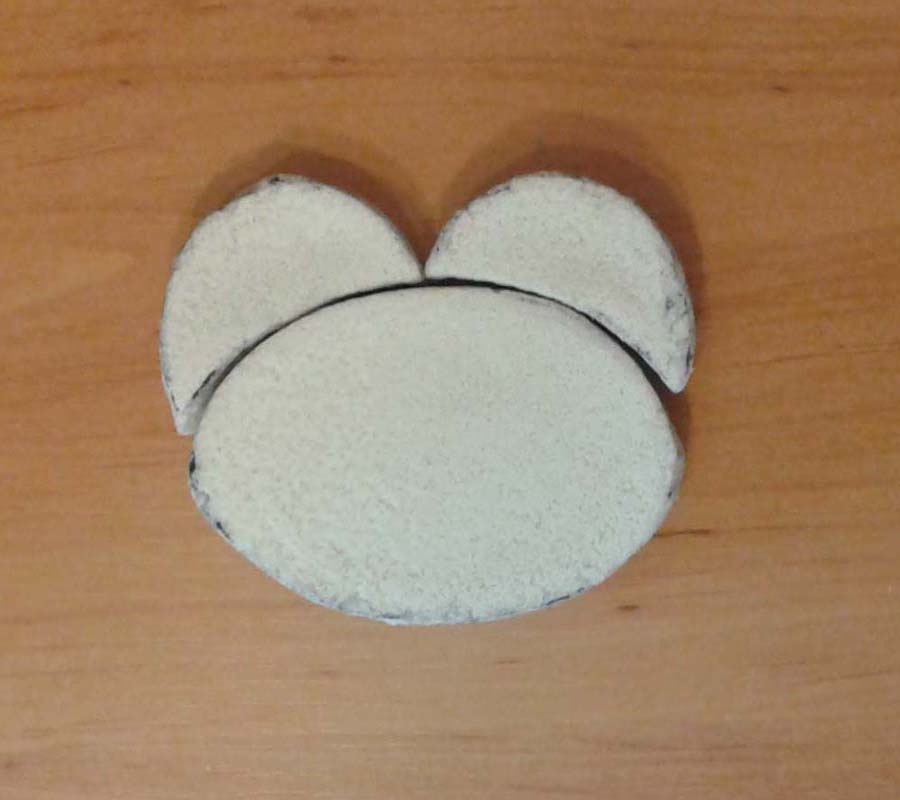

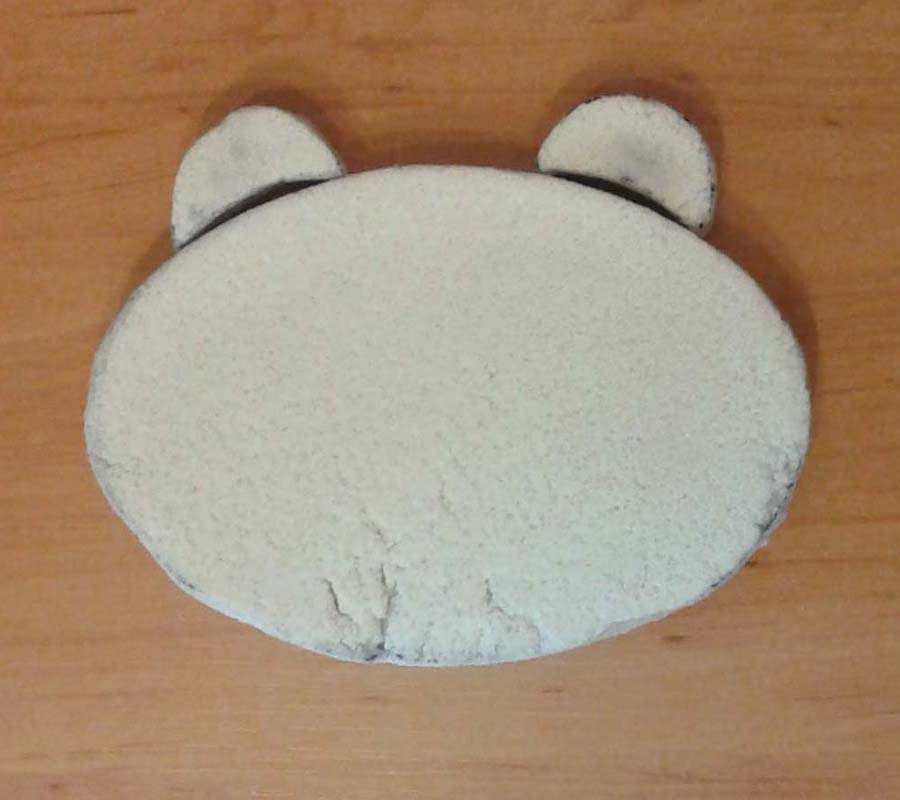

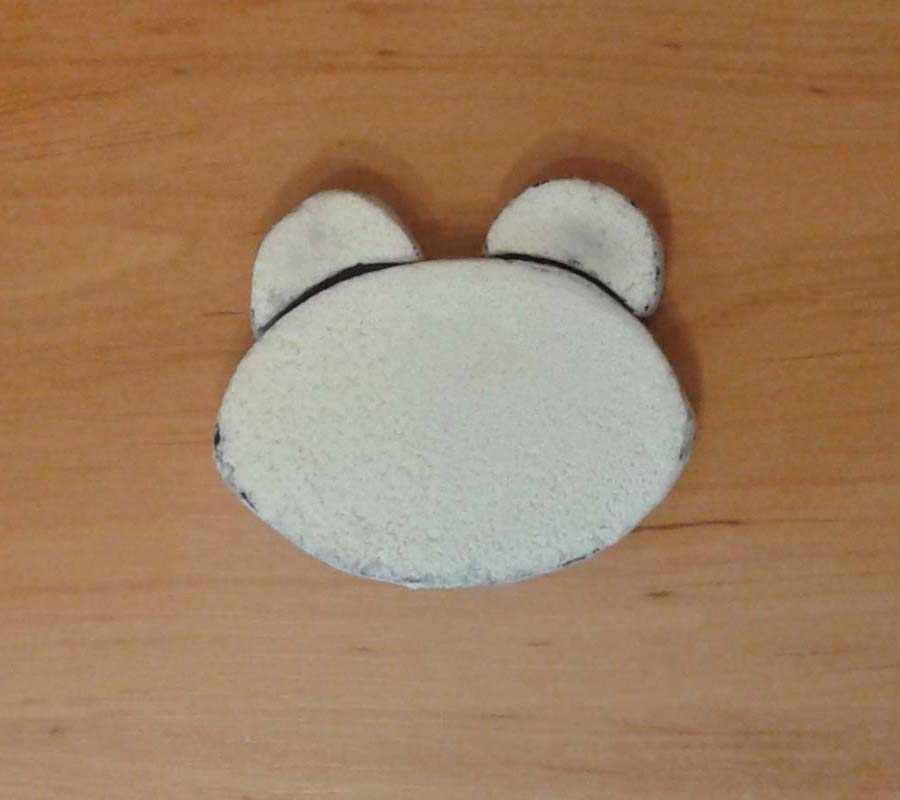


Fig. 2. Combinations of chest and breast versions.


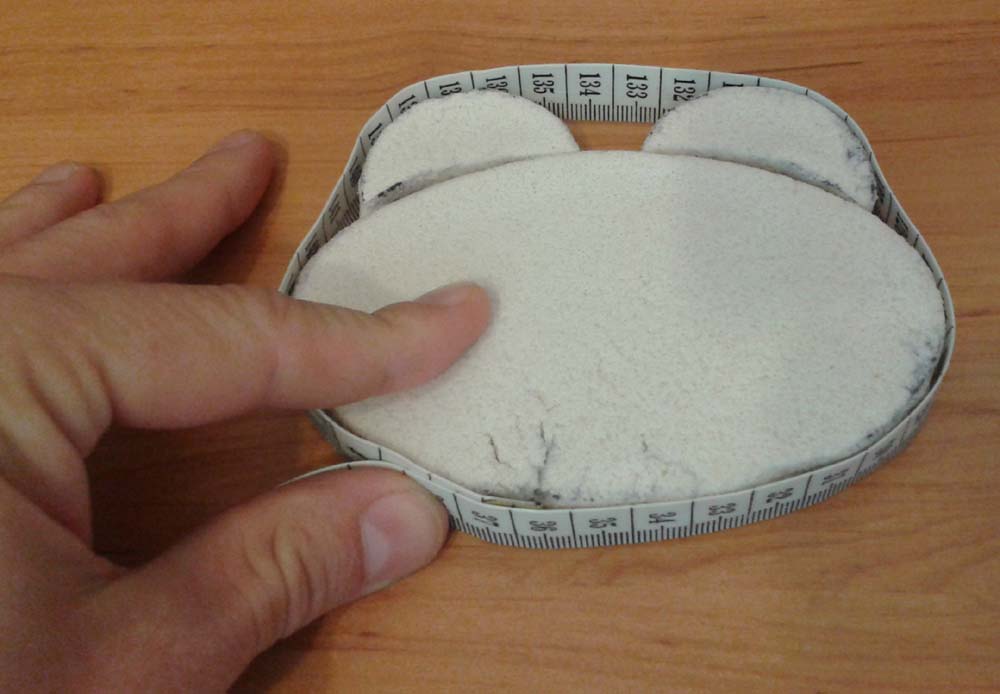
Fig. 3. Measurement of female body models.


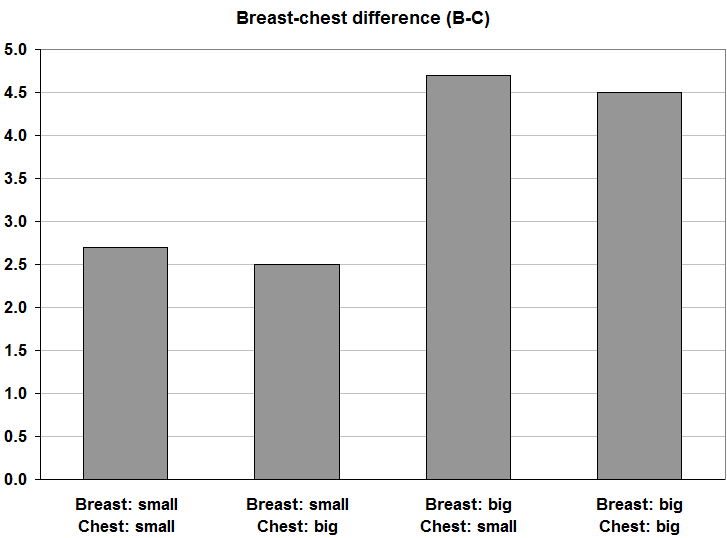
Fig. 4. Breast-chest girth difference as dependent on chest and breast size.


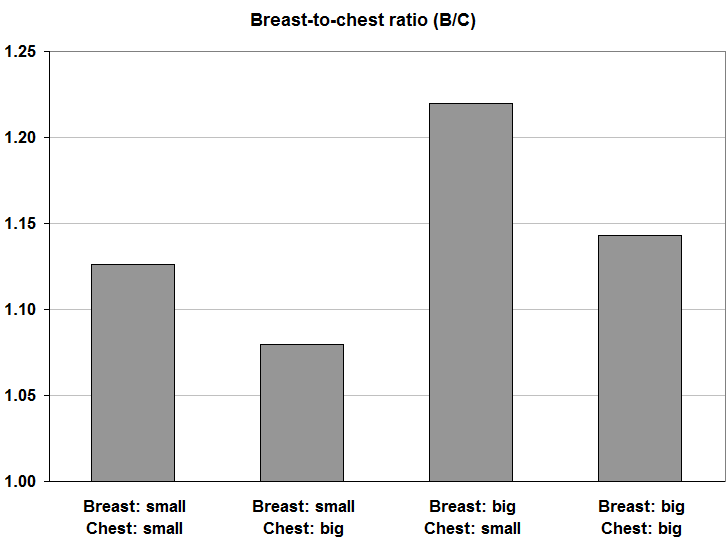
Fig. 5. Breast-to-chest girth ratio as dependent on chest and breast size.


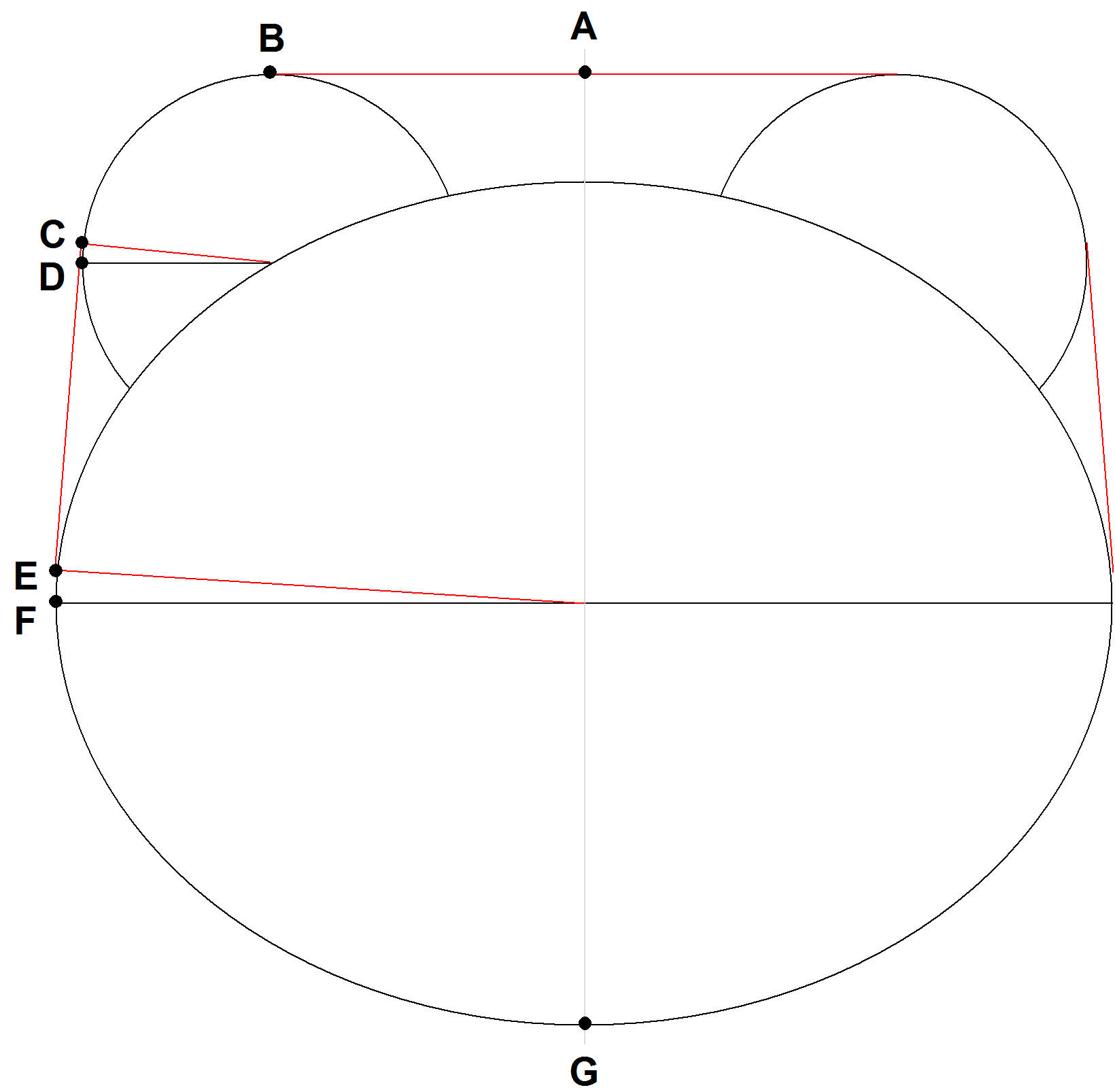


Fig. 6. Estimation of trunk circumference measured with a measuring tape at the level of breast.

Half of the girth was calculated as: AB + BD + CE – CD + EF + FG, where AB, CE, CD, and EF are segment lengths (CE being tangent to the breast and chest surface), and BC and FG are arc lengths, the fourth of the circle and the fourth of the ellipse, respectively.

The Peano’s formula for ellipse perimeter was applied: π × [1.5 × (width/2 + depth/2) – √(width/2 × depth/2)].


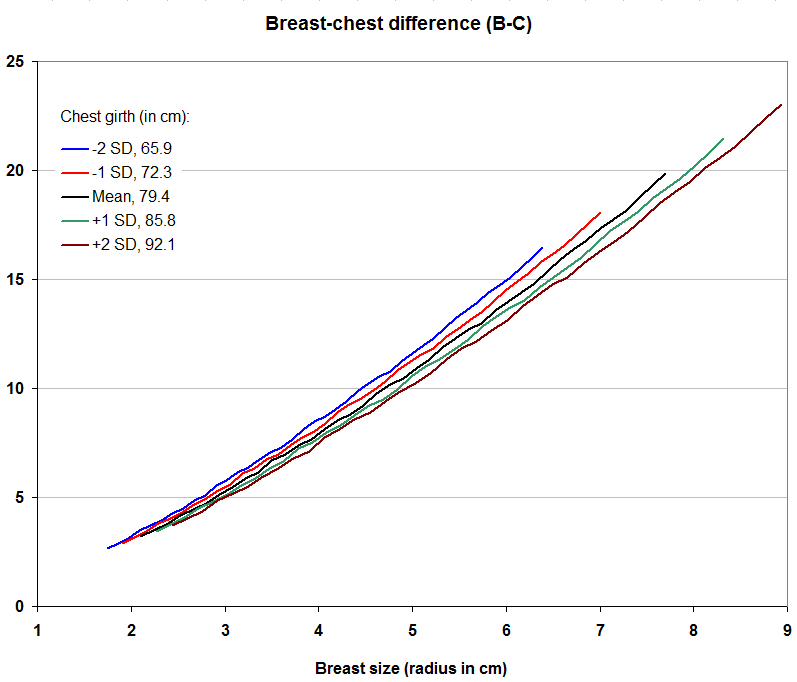
Fig. 7. Dependence of breast-chest difference on breast size for 5 variants of chest size.


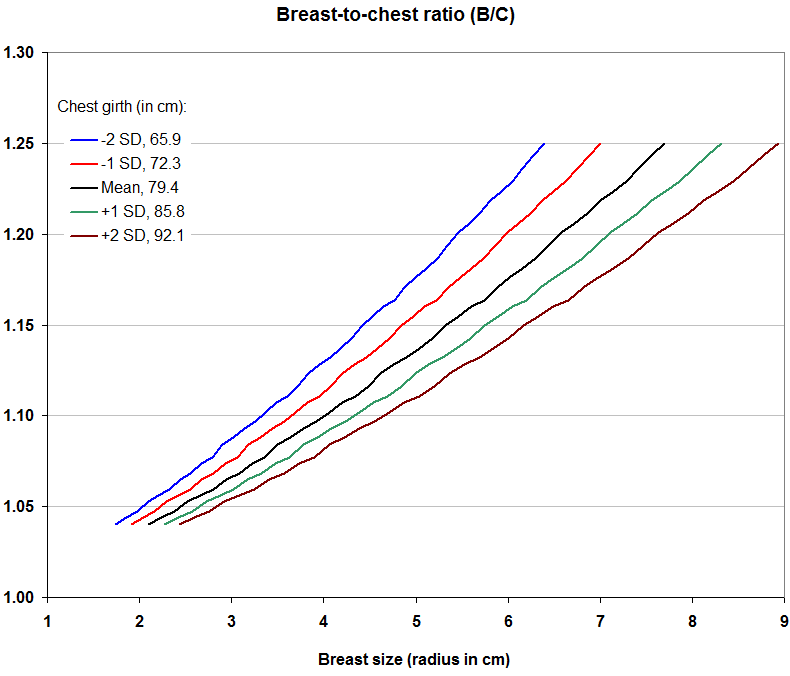
Fig. 8. Dependence of breast-to-chest ratio on breast size for 5 variants of chest size.

*Perception of women in relation to breast and hip size, and hair color – supplementary data*


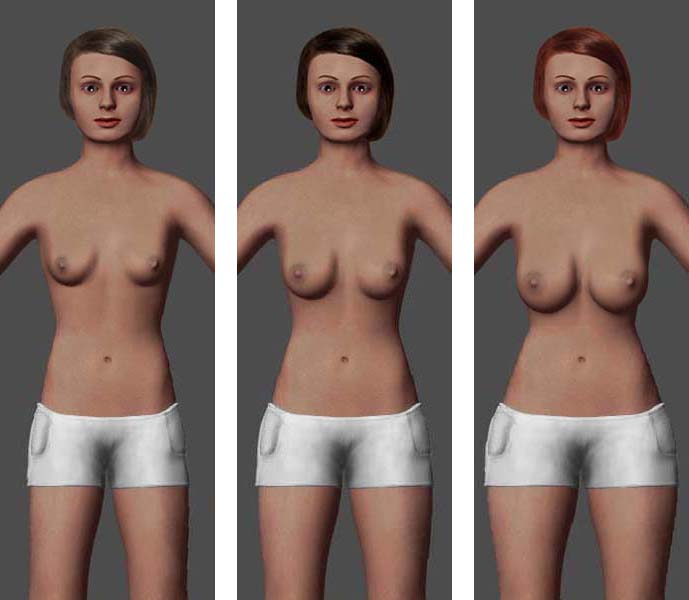
Figure S1. Example digital female figures varying in breast size, hip width, and hair color. Altogether, 27 figure versions were manufactured: 3 breast sizes × 3 hip widths × 3 hair colors.

Table S1. Means for evaluations of ten characteristics in a female digital model varying in three morphological traits (breast size, hip width, and hair color), and Tukey test’s *p*-values for differences between variants of these traits.

(a) Breast size

|  | Breast size | | |  | *p*-values for differences | | |
| --- | --- | --- | --- | --- | --- | --- | --- |
|  | Small | Average | Big |  | Small vs. Average | Small vs. Big | Average vs. Big |
| Physical attractiveness | 3.93 | 5.00 | 5.11 |  | .000 | .000 | .603 |
| Sexual desire | 3.60 | 4.61 | 4.97 |  | .000 | .000 | .004 |
| Sociosexual orientation | 3.82 | 4.25 | 4.64 |  | .000 | .000 | .003 |
| Reproductive efficiency | 4.52 | 5.16 | 5.49 |  | .000 | .000 | .012 |
| Lactational efficiency | 3.59 | 5.15 | 6.10 |  | .000 | .000 | .000 |
| Marital attractiveness | 4.21 | 5.32 | 5.25 |  | .000 | .000 | .847 |
| Sexual attractiveness | 4.01 | 5.26 | 5.70 |  | .000 | .000 | .007 |
| Faithfulness | 4.98 | 4.86 | 4.56 |  | .572 | .001 | .035 |
| Intelligence | 5.08 | 5.08 | 4.66 |  | 1.000 | .000 | .000 |
| Diligence | 5.02 | 4.92 | 4.76 |  | .666 | .044 | .276 |

(b) Hip width

|  | Hip width | | |  | *p*-values for differences | | |
| --- | --- | --- | --- | --- | --- | --- | --- |
|  | Narrow | Average | Wide |  | Narrow vs. Average | Narrow vs. Wide | Average vs. Wide |
| Physical attractiveness | 4.55 | 5.18 | 4.31 |  | .000 | .102 | .000 |
| Sexual desire | 4.23 | 4.78 | 4.18 |  | .000 | .890 | .000 |
| Sociosexual orientation | 4.23 | 4.28 | 4.20 |  | .906 | .979 | .809 |
| Reproductive efficiency | 3.95 | 5.06 | 6.16 |  | .000 | .000 | .000 |
| Lactational efficiency | 4.74 | 4.95 | 5.15 |  | .095 | .000 | .087 |
| Marital attractiveness | 4.73 | 5.30 | 4.75 |  | .000 | .984 | .000 |
| Sexual attractiveness | 5.00 | 5.35 | 4.62 |  | .036 | .025 | .000 |
| Faithfulness | 4.73 | 4.66 | 5.00 |  | .799 | .058 | .010 |
| Intelligence | 4.91 | 5.07 | 4.83 |  | .257 | .711 | .048 |
| Diligence | 4.85 | 4.94 | 4.91 |  | .713 | .841 | .973 |

(c) Hair color

|  | Hair color | | |  | *p*-values for differences | | |
| --- | --- | --- | --- | --- | --- | --- | --- |
|  | Light | Dark | Red |  | Light vs. Dark | Light vs. Red | Dark vs. Red |
| Physical attractiveness | 4.62 | 4.74 | 4.68 |  | .563 | .896 | .834 |
| Sexual desire | 4.32 | 4.37 | 4.49 |  | .874 | .269 | .543 |
| Sociosexual orientation | 4.22 | 3.99 | 4.49 |  | .123 | .045 | .000 |
| Reproductive efficiency | 5.06 | 4.95 | 5.17 |  | .586 | .628 | .137 |
| Lactational efficiency | 5.00 | 4.79 | 5.06 |  | .080 | .833 | .017 |
| Marital attractiveness | 5.02 | 4.89 | 4.86 |  | .607 | .446 | .963 |
| Sexual attractiveness | 5.05 | 4.92 | 5.00 |  | .619 | .932 | .832 |
| Faithfulness | 4.90 | 4.73 | 4.77 |  | .353 | .509 | .961 |
| Intelligence | 4.92 | 4.99 | 4.91 |  | .759 | .999 | .735 |
| Diligence | 4.91 | 4.89 | 4.90 |  | .852 | .881 | .970 |

#### *References*

Batogowska A, Słowikowski J, 1994, Atlas antropometryczny dorosłej ludności Polski dla potrzeb projektowania. Prace i Materiały IWP, 149, Warszawa [in Polish]

Brown N, White J, Milligan A, Risius D, Ayres B, Hedger W, Scurr J, 2012, The relationship between breast size and anthropometric characteristics. Am J Hum Biol, 24, 158-164

Garver-Apgar CE, Eaton MA, Tybur JM, Emery Thompson M, 2011, Evidence of intralocus sexual conflict: Physically and hormonally masculine individuals have more attractive brothers relative to sisters. Evol Hum Behav, 32, 423-432

Grillot RL, Simmons ZL, Lukaszewski AW, Roney JR, 2014, Hormonal and morphological predictors of women’s body attractiveness. Evol Hum Behav, 35, 176-183

Jansen LA, Backstein RM, Brown MH, 2014, Breast size and breast cancer: a systematic review. J Plast Reconstr Aesthet Surg, 67, 1615-1623

Jasieńska G, Ziomkiewicz A, Ellison PT, Lipson SF, Thune I, 2004, Large breasts and narrow waists indicate high reproductive potential in women. Proc R Soc Lond B, 271, 1213-1217

Kayar R, Civelek S, Cobanoglu M, Gungor O, Catal H, Emiroglu M, 2011, Five methods of breast volume measurement: a comparative study of measurements of specimen volume in 30 mastectomy cases. Breast Cancer, 27, 5, 43-52

Lynn M, 2009, Determinants and consequences of female attractiveness and sexiness: Realistic tests with restaurant waitresses. Arch Sex Behav, 38, 737-745

Ray JG, Mohllajee AP, van Dam RM, Michels KB, 2008, Breast size and risk of type 2 diabetes mellitus. Can Med Assoc J, 178, 289-295

1. Although Garver-Apgar et al. (2011) applied the formula of (Breast – Chest) / Chest, it is mathematically equivalent to Breast / Chest – Chest / Chest, or Breast / Chest – 1, which is effectively the Breast-to-Chest girth ratio. [↑](#footnote-ref-2)
2. Data for chest size variation was taken from Batogowska et al. (1994). Variation of breast size was determined on the basis of the photograph of the topless woman and body measurements taken on participants of Study 2 (see the main text). [↑](#footnote-ref-3)
